# Long Non-Coding RNAs as Potential Diagnostic and Prognostic Biomarkers in Breast Cancer: Progress and Prospects

**DOI:** 10.3389/fonc.2021.710538

**Published:** 2021-08-30

**Authors:** Cuicui Lu, Duncan Wei, Yahui Zhang, Peng Wang, Wen Zhang

**Affiliations:** ^1^Department of Pharmacy, Shandong Provincial Hospital Affiliated to Shandong First Medical University, Jinan, China; ^2^Department of Pharmacy, The First Affiliated Hospital of Medical College of Shantou University, Shantou, China; ^3^Department of Pharmacy, The First Affiliated Hospital of Shandong First Medical University, Jinan, China

**Keywords:** long non-coding RNA, breast cancer, biomarker, diagnosis, prognosis

## Abstract

Breast cancer is the most common malignancy among women worldwide, excluding non-melanoma skin cancer. It is now well understood that breast cancer is a heterogeneous entity that exhibits distinctive histological and biological features, treatment responses and prognostic patterns. Therefore, the identification of novel ideal diagnostic and prognostic biomarkers is of utmost importance. Long non-coding RNAs (lncRNAs) are commonly defined as transcripts longer than 200 nucleotides that lack coding potential. Extensive research has shown that lncRNAs are involved in multiple human cancers, including breast cancer. LncRNAs with dysregulated expression can act as oncogenes or tumor-suppressor genes to regulate malignant transformation processes, such as proliferation, invasion, migration and drug resistance. Intriguingly, the expression profiles of lncRNAs tend to be highly cell-type-specific, tissue-specific, disease-specific or developmental stage-specific, which makes them suitable biomarkers for breast cancer diagnosis and prognosis.

## Introduction

Breast cancer is a major public health dilemma on a global scale. Inherited and acquired genetic as well as epigenetic alterations have been extensively demonstrated as the driving events of breast cancer (1). Breast cancer is a heterogeneous entity, and different subtypes exhibit distinctive histological and biological features, treatment responses and prognostic patterns (2). Despite substantial advancement in early detection and management, breast cancer remains the second-leading cause of cancer-related death among women worldwide (3). Moreover, advanced or metastatic breast cancer is almost incurable by current systemic treatment options (4). As such, one key challenge in breast cancer therapy is to identify novel reliable diagnostic and prognostic biomarkers.

Only approximately 2% of the human genome is composed of protein-coding transcripts, indicating that the majority of transcripts are non-coding RNAs (ncRNAs) (5). NcRNAs are broadly divided into small ncRNAs (20~200 nucleotides) and long ncRNAs (lncRNAs, >200 nucleotides) (6). LncRNAs were previously considered “transcriptional noise” due to the lack of a significant open reading frame (7). However, increasing evidence indicates that lncRNAs are involved in different biological and pathological processes, including cell apoptosis, differentiation and autophagy (8). Over the last decade, high-throughput and next-generation sequencing technologies have allowed the study of RNAs in an unbiased manner. These technological advances contribute to an explosion of genomic information and increase the ability to identify novel lncRNAs.

More importantly, accumulating evidence suggests that lncRNAs are involved in various human cancers, including breast cancer. Approximately 1,900 lncRNAs are dysregulated in breast cancer (9), and their levels may be associated with distinct clinical outcomes. Dysregulated lncRNAs can act as oncogenes or tumor suppressors to control breast cancer pathophysiology and should be investigated to obtain a better understanding of their roles in breast cancer biology and determine their suitability as diagnostic and prognostic biomarkers. Therefore, this study aims to review the current knowledge about lncRNAs and evaluate their potential roles as molecular markers in breast cancer.

## Breast Cancer

Breast cancer is the most common cancer among women worldwide, excluding non-melanoma skin cancer (10). In 2018, approximately 2.1 million cases of breast cancer were newly diagnosed, and approximately 626,679 patients died that same year (10). The well-established risk factors for breast cancer are race, family history of cancer, genetic susceptibility, modifiable exposures, environmental factors and unhealthy lifestyles (11). The incidence of breast cancer varies worldwide and is higher in high-income countries than in low-income countries. However, the death rate in lower-income regions is higher due to the lack of early diagnosis and limited access to treatment (12).

Breast cancer is a complex, heterogeneous disease, and different subtypes have distinct clinical presentations and therapeutic responses. Breast cancer is defined into 3 clinically relevant subtypes according to estrogen receptor (ER) expression, progesterone receptor (PR) expression and human epidermal growth factor 2 (ERBB2; formerly HER2) gene amplification: ER-positive/PR-positive, HER2/ERBB2-positive, and triple-negative (lacking expression of all three molecular markers) (2). Triple-negative breast cancer is an aggressive subtype and accounts for 15% of breast tumors (13). It has been well established that triple-negative tumors have a relatively high mitotic activation index, prominent lymphocytic infiltrate, high incidence of distant relapse, and poor clinical outcomes (14). Breast cancer is staged I-IV, and stage IV has distant metastases at diagnosis. Metastatic breast cancer remains essentially incurable, and the therapeutic goals are symptom palliation and prolonging life. The median overall survival (OS) of stage IV breast cancer patients for the triple-negative and HER2/ERBB2-positive subtypes was approximately 1 year and 5 years, respectively (15). As such, detecting breast cancer at an early stage is of paramount importance.

## Discovery, Classification, and Function of LNCRNAS

LncRNAs are commonly defined as RNA transcripts larger than 200 nucleotides. Once considered junk DNA, lncRNAs have recently attracted wide attention as crucial regulators in a diverse array of biological processes. Over the past decade, technical advancements in high-throughput sequencing have greatly streamlined the process of identifying all forms of RNAs. To date, more than 58,000 lncRNAs have been identified, and approximately 30,000 lncRNA transcripts have been curated in GENCODE v29 (16).

Like other RNAs, lncRNAs mainly consist of four core nucleotides (17). LncRNAs exhibit the same characteristics as mRNA transcripts, i.e., they are RNA polymerase II–transcribed, 5nscribedrase II–aracteristics as mR (18). Almost all lncRNAs are localized in the cell nucleus, but some lncRNAs can be exported to the cytoplasm (19). With respect to their genomic location, lncRNAs can be classified into intronic, intergenic, sense, antisense and bidirectional loci (6). Unlike the well-studied miRNAs, lncRNA homologs exhibit weak or untraceable primary sequence conservation (20). Importantly, lncRNAs have evolutionarily conserved promoters, suggesting the importance of lncRNA regulation. Furthermore, compared to small ncRNAs, lncRNAs have highly conserved secondary and tertiary structures, which are considered their major functional units (21).

**Figure 1:**
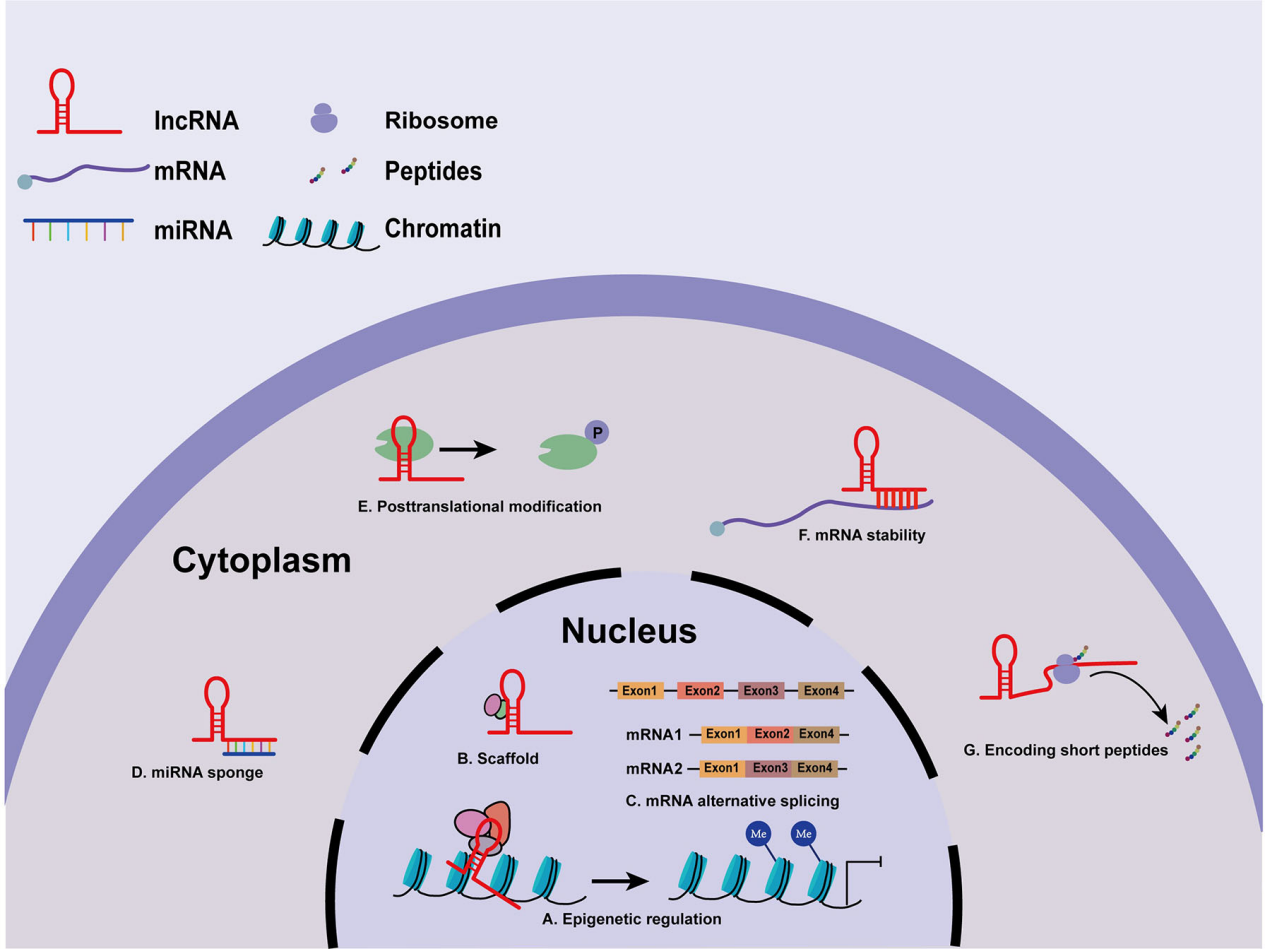
Molecular mechanisms of lncRNAs. **(A)** LncRNAs modulate histone modification and chromatin remodeling. **(B)** LncRNAs serve as scaffolds for various proteins. **(C)** Nuclear lncRNAs participate in mRNA processing. **(D)** LncRNAs act as miRNA sponges or ceRNAs in regulating miRNAs. **(E)** LncRNAs regulate the stability and posttranslational modifications of some proteins. **(F)** lncRNAs stabilize mRNAs. **(G)** lncRNAs encode functional small peptides.

LncRNAs have been identified as indispensable regulatory elements in multiple cell processes, such as chromatin modification and transcriptional and post-transcriptional regulation (22). There is accumulating evidence that the biological functions of lncRNAs depend strictly on their subcellular location (23). Nuclear lncRNAs can function as cis- and trans-acting elements to modulate chromatin remodeling (24). Some lncRNAs may recruit DNA or chromatin regulatory complexes to regulate the epigenetic silencing or activation of target loci by altering histone modifications or DNA methylation patterns (25) **(** **Figure 1A** **)**. Intriguingly, lncRNAs have dynamic and flexible biophysical structures, which confer lncRNAs the ability to serve as scaffolds and allow the assembly of various proteins (26) **(** **Figure 1B** **)**. Besides, nuclear lncRNAs can also modulate messenger RNA (mRNA) alternative spicing (27) **(** **Figure 1C** **)**. In the cytoplasm, lncRNAs may act as miRNA sponges or competitive endogenous RNAs (ceRNAs) to prevent miRNA binding with target mRNAs, hence regulating the expression of downstream target genes at the post-transcriptional level (28) **(** **Figure 1D** **)**. Recent studies have shown that lncRNAs interact with some proteins to regulate their stability and posttranslational modifications (29) **(** **Figure 1E** **)**. Furthermore, cytoplasmic lncRNAs transcribed from enhancer regions can stabilize mRNAs by recruiting specific proteins (30) **(** **Figure 1F** **)**. More interestingly, some lncRNAs even hold the potential to encode functional small peptides (31) **(** **Figure 1G** **)**. Collectively, lncRNAs exert regulatory roles by directly or indirectly interacting with DNA, RNA, or proteins, and the functions of lncRNAs are still under investigation.

Recent studies have shed light on the regulatory effects of lncRNAs in human cancers. Importantly, some aberrant lncRNAs hold the promise to serve as ideal biomarkers in certain cancers. Here, main lncRNAs with diagnostic and prognostic values in breast cancer are summarized in **Table 1**.

**Table 1:** Summary of breast cancer-associated lncRNAs.

LncRNAs	Expression status	Oncogene/tumor suppressor	Cell processes	Clinical outcomes	References
MALAT1	Upregulated	Oncogene	Proliferation, invasion, migration, recurrence, drug resistance and angiogenesis	Unfavorable OS, DFS and RFS	(32–47)
NEAT1	Upregulated	Oncogene	Proliferation, invasion and metastasis	Tumor volume, lymph node metastasis and poor prognosis	(48–56)
H19	Upregulated	Oncogene	Proliferation, metastasis, invasion	Shorter OS and DFS	(57–64)
AFAP1-AS1	Upregulated	Oncogene	Proliferation, anti-apoptosis, migration and invasion	Greater tumor volume, advanced TNM stage, lymph node metastasis and distant metastasis	(65–72)
HOTAIR	Upregulated	Oncogene	Proliferation, anti-apoptosis, migration and invasion	Advanced tumor stage, enhanced metastasis and unfavorable prognosis	(73–79)
ROR	Upregulated	Oncogene	Cell growth, migration, invasiveness and drug resistance	Poor prognosis	(80–85)
ANRIL	Upregulated	Oncogene	Proliferation	Increased tumor size, advanced TNM stage and unfavorable prognosis	(86–91)
BC200	Upregulated	Oncogene	Proliferation, migration and invasion	Poor prognosis	(92–95)
SPRY4-IT1	Upregulated	Oncogene	Proliferation, invasion and metastasis	Increased tumor size, high TNM stage, lymph node metastasis and unfavorable prognosis	(96–99)
UCA1	Upregulated	Oncogene	Proliferation, invasion and metastasis	Increased lymph node metastasis and shorter OS	(100–104)
ATB	Upregulated	Oncogene	Drug resistance, anti-apoptosis, proliferation, invasion and metastasis	More nodal metastasis, advanced clinical stage and unfavorable prognosis	(105–109)
PVT1	Upregulated	Oncogene	Proliferation, invasion and migration	More lymph node metastasis, increased distant metastasis, advanced TNM stage, poor differentiation grade and unfavorable prognosis	(110–115)
CCAT1	Upregulated	Oncogene	Radio resistance, proliferation, invasion and metastasis	Histological grade, TNM staging and lymph metastasis, and poor prognosis	(116–119)
CCAT2	Upregulated	Oncogene	Proliferation, invasion and metastasis	Greater tumor volume, higher TNM grades, advanced clinical stage and a poor OS	(120–124)
TINCR	Upregulated	Oncogene	Proliferation, anti-apoptosis, migration and invasion	unfavorable prognosis	(125–128)
MEG3	Downregulated	Tumor suppressor	Proliferation, anti-apoptosis, metastasis and invasion	Advanced TNM stage, lymph node metastasis and differentiation grade	(129–137)
XIST	Downregulated	Tumor suppressor	Proliferation, migration, invasion and anti-apoptosis	Larger tumor volume, increased lymphatic metastasis, advanced tumor stage and unfavorable prognosis	(138–144)
PTENP1	Downregulated	Tumor suppressor	Proliferation, colony formation, migration, invasion and anti-apoptosis	Advanced TNM stage and worse OS	(145–149)

## Oncogenic LNCRNAS

### Metastasis-Associated Lung Adenocarcinoma Transcript 1

MALAT1, located in the nucleus, is approximately 8,000 nucleotides in length. MALAT1 is among the most conserved and extremely abundant lncRNAs in different tissues, suggesting that it may have vital biological functions (32, 33). MALAT1 was initially identified as a tumor promoter in non-small cell lung cancer (34). To date, MALAT1 overexpression has been shown in multiple human cancers, such as ovarian, bladder and colorectal cancers (35–37). It has been demonstrated that serum MALAT1 levels in breast cancer patients are markedly higher than those in patients with benign breast disease (38). Huang et al. indicated that the expression of MALAT1 was elevated in breast cancer patients compared to healthy cases. Moreover, silencing of MALAT1 significantly hindered angiogenesis *via* upregulation of miR-145 expression (39). It has also been found that MALAT1 promotes epithelial-mesenchymal transition (EMT) mainly by regulating the miR-204/ZEB2 axis (40). Another study demonstrated that MALAT1 could enhance the proliferation and invasion of breast cancer cells by altering the histone 3 lysine 4 (H3K4) epigenotype to activate the EEF1A1 promoter. Downregulation of MALAT1 expression may significantly reduce the promoter activity of EEF1A1, suggesting a novel MALAT1-mediated epigenetic mechanism of EEF1A1 regulation (41). Zhao et al. indicated that a high concentration of 17β-estradiol inhibited the proliferation, invasion and metastasis of breast cells *via* downregulation of MALAT1 expression (42). In breast cancer, early postoperative fever indicates unfavorable clinical outcomes. Li et al. demonstrated that overexpression of MALAT1 in breast cancer patients with early postoperative fever was significantly related to inflammatory responses and lung metastasis (43). In addition, Wang et al. indicated that elevated MALAT1 levels were inversely correlated with OS in invasive ductal carcinoma (44). Furthermore, a meta-analysis showed that high expression of MALAT1 in breast cancer is correlated with unfavorable disease-free survival (DFS) and recurrence-free survival (RFS) (45). In recent years, accumulating evidence has shown that the aberrant expression of MALAT1, especially in serum/plasma, may serve as a suitable biomarker in various human cancer entities (46, 47).

### Nuclear Enriched Abundant Transcript 1

NEAT1 has two variants (NEAT1-1 and NEAT1-2), and it is indispensable for paraspeckle integrity (150). In the last few years, several studies indicating the involvement of NEAT1 in breast cancer have been published, and NEAT1 is closely related to different miRNAs. Jiang et al. indicated that high levels of NEAT1 in diverse breast cancer cell lines were linked to aggressive progression and unfavorable prognosis. They also found that miR-448, an inhibitor of cancer cell growth, was inhibited by NEAT1 and consequently led to increased expression of zinc-finger E-box binding protein 1 (ZEB1), an oncogene (48). Zhang et al. demonstrated that ectopic expression of NEAT1 was related to tumor volume and lymph node metastasis, while silencing of NEAT1 expression resulted in decreased proliferation and migration in breast cancer cell lines (49). In addition, overexpression of NEAT1 leads to the downregulated expression of miR-133b, which is a known inhibitor of tumorigenesis, consequently resulting in enhanced migration and invasion (50). Ke et al. indicated that downregulation of NEAT1 expression by miR-548 could abrogate proliferation and induce apoptosis in breast cancer. They also found that fused in sarcoma (FUS), a nuclear RNA binding protein, directly interacted with NEAT1, and the role of NEAT1 in cancer cell survival was mediated by FUS (51). Enhancer of zest homolog 2 (EZH2), known as a molecular marker of aggressive malignancies, is a target of miR-101. Qian et al. showed that NEAT1 promoted the growth of breast cancer cells *via* miR-101-dependent EZH2 regulation (52). In addition, NEAT1 could increase the expression of high mobility group AT-hook 2 (HMGA2) by sponging miR-211, thereby enhancing the invasiveness of breast cancer cells (53). High levels of NEAT1, miR-21, and RRM2 have been observed in different breast cancer cell lines, and their elevated levels correlate with poor clinical outcomes, suggesting that the NEAT1/miR-21-RRM2 signaling axis contributes to breast cancer development (54). Moreover, NEAT1 acts as a sponge for 146b-5p to promote the proliferation, migration, and metastasis of breast cancer cells (55). Zhou et al. showed that NEAT1 could coordinate various miRNAs in different breast cancer subtypes and thus exert diverse regulatory functions (56). In conclusion, these data revealed various mechanisms of NEAT1 in the regulation of breast cancer and suggested that NEAT1 might function as a potential biomarker in breast cancer.

### H19

H19, an imprinted gene, is located on chromosome 11p15.5 (151). H19 is abundantly expressed during embryogenesis, and the expression of H19 is repressed upon birth, except for basal expression in adult tissues, such as lung, skeletal muscle and mammary gland (152). The first evidence that H19 has a pivotal role in breast cancer was provided by Adriaenssens et al., who found that H19 overexpression was significantly associated with ER/PR status and tumor progression (57). Matouk et al. found that H19 suppressed the expression of E-cadherin, a representative inhibitor of EMT, and promoted metastasis *via* regulation of Slug in breast cancer (58). H19 functions as a molecular sponge of miR‐152 to upregulate the expression of the DNA methyltransferase DNMT1, thus facilitating the proliferation and invasiveness of breast cancer cells (59). It has also been demonstrated that H19 can sponge miR-200b/c and let-7b differently to enhance EMT and mesenchymal-epithelial transition (MET) (60). Additionally, downregulation of H19 expression could result in S-phase arrest of breast cancer cells, suggesting its role in regulating cell cycle progression (61). H19 could serve as a precursor for miR-675, which is encoded by the exon of H19. Vennin et al. found that high expression of H19 upregulated miR-675 expression, negatively regulating E3 ubiquitin ligases (c-Cbl and Cbl-b) to enhance the aggressiveness of breast cancer cells (62). Zhang et al. showed that high plasma H19 levels were correlated with ER/PR status and lymph node metastasis (63). Moreover, elevated levels of H19 have been significantly associated with unfavorable OS and DFS, particularly in the triple-negative subtype (64). A meta-analysis showed that dysregulated H19 expression correlated with poor differentiation, high tumor stage, early distant metastasis, and lymph node involvement in multiple cancers (153). In sum, current evidence establishes H19 as a potential breast cancer biomarker.

### Actin Filament Associated Protein 1 Antisense RNA1

AFAP1-AS1 is 6,810 bp long and is located on human chromosome 4p16.1 (154). Further studies have shown that AFAP1-AS1 expression is markedly upregulated in breast cancer tissues and cell lines and predicts poor clinical outcomes (65). In addition, AFAP1-AS1 overexpression in cancers correlates with greater tumor volume, advanced tumor-node-metastasis (TNM) staging, lymph node metastasis and distant metastasis (65). Intriguingly, elevated levels of AFAP1-AS1 are more common in triple-negative breast cancer (66). Previous experiments suggested that AFAP1-AS1 could promote tumorigenesis by interfering with AFAP1 expression (67). However, Dianatpour et al. demonstrated that high levels of AFAP1-AS1 had no regulatory effect on AFAP1 expression in breast cancer patients (68). Consistent with this study, Ma et al. indicated that silencing of AFAP1-AS1 exerted no effects on AFAP1 expression or actin filament integrity (69). Such discrepancies among different cancers need to be further elucidated. Ki-67, a nuclear antigen, is not expressed in quiescent cells (70). Downregulation of AFAP1-AS1 expression was detected in all of the Ki-67-negative samples, suggesting that AFAP1-AS1 might be implicated in cell proliferation (68). Moreover, AFAP1-AS1 regulates the wnt/β-catenin pathway, facilitating the expression of c-Myc and EMT-associated transcription factors to promote tumorigenesis and induce EMT (66). Furthermore, AFAP1-AS1 directly binds to miR-497-5p to upregulate the expression of Septin 2, a well-known oncogene. Depletion of AFAP1-AS1 inhibits proliferation and migration and induces apoptosis in breast cancer (71). In triple-negative breast cancer, AFAP1-AS1 sponges miR-154 to coordinate the expression of MutT homolog-1, which in turn induces cellular proliferation and invasion (72). Hence, the dysregulated expression of AFAP1-AS1 and its molecular mechanisms identify it as a putative biomarker and actionable target in breast cancer.

### HOX Transcript Antisense RNA

HOTAIR is a 2,158-bp lncRNA located on human chromosome 12q13.13 between the HOXC11 and HOXC12 genes (155). Ectopic HOTAIR expression has been implicated in a variety of cancers, such as pancreatic, colorectal and non-small-cell lung cancers (156–158). HOTAIR expression seems to be elevated in cancer tissues compared to paired non-cancerous tissues, and high expression of HOTAIR has been associated with an enhanced proliferation rate, advanced tumor stage, elevated risk of metastasis, and unfavorable prognosis (73, 74). Gupta et al. indicated that HOTAIR expression was upregulated in primary breast tumors and metastases and that dysregulation of HOTAIR in primary tumors correlated with metastasis and poor prognosis (75). On the other hand, treatment with transforming growth factor-β1 (TGF-β1) upregulates the expression of HOTAIR and contributes to EMT. TGF-β1-induced EMT is reversed by HOTAIR knockdown, suggesting that the effect of TGF-β1 on EMT is, at least partly, mediated through HOTAIR (76). Mechanistically, depletion of HOTAIR inhibits the growth, invasion and migration of breast cancer cells through downregulation of p53 expression (77). In addition, HOTAIR sequesters miR-206 to enhance the expression of Bcl-w, an anti-apoptotic protein, thereby promoting the proliferation of breast cancer cells (78). Moreover, HOTAIR could recruit polycomb repressive complex 2 (PRC2), known as a transcriptional corepressor, to facilitate epigenetic gene silencing (75). However, another study demonstrated that the oncogenic role of HOTAIR in breast cancer cells may be independent of PRC2. Instead, the recruitment of PRC2 seemed to be a consequence of gene silencing (79). These contradictory findings on HOTAIR have caused confusion about its role in breast cancer. Hence, further studies are needed to elucidate the interaction between HOTAIR and PRC2. Overall, the involvement of HOTAIR in these signaling pathways contributes to the progression of breast cancer, and HOTAIR might be utilized as a new predictive and prognostic biomarker in breast cancer.

### Regulator of Reprogramming

ROR is a 2.6-kb intergenic transcript located on chromosome 18q21.31 (159). ROR was initially discovered as a promoter of the reprogramming process, and it was shown to contribute to the maintenance of pluripotent and embryonic stem cells *via* inhibition of cellular stress signaling pathways (159). Elevated ROR expression has been detected across cancer cell lines (160). Recent studies have shown that ROR promotes EMT in various cancers (161, 162). Accordingly, ROR overexpression induces EMT and promotes cell growth, migration and invasiveness in breast cancer (80–82). Functionally, ROR regulates the TGF-β pathway to promote breast cancer progression, whereas suppression of ROR inhibits tumor growth (80). In addition, ROR acts as a ceRNA for miR-205 to upregulate the expression of a miR-205 target gene, the EMT inducer ZEB2 (81). Zhou et al. showed that ROR sponged miR-194-3p and upregulated the expression of a miR-194-3p target, the methyl-CpG-binding protein 2 (MECP2) gene, to decrease the sensitivity of breast cancer cells to rapamycin (82). Moreover, ROR activated the MAPK/ERK pathway and upregulated the expression of dual specificity phosphatase 7 (DUSP7), an ERK-specific phosphatase, thereby facilitating estrogen-independent proliferation of breast cancer cells (83). Furthermore, silencing ROR reversed gemcitabine-induced apoptosis and autophagy in MDA-MB-231 cell lines. Mechanistically, ROR decreased acetylated histone H3 at the miR-34a promoter and resulted in increased expression of autophagy-related genes and decreased expression of p62 (84). ROR polymorphisms could influence cancer susceptibility. For instance, Luo et al. indicated that the TT genotype of ROR rs4801078 correlated with elevated ROR mRNA levels and an increased risk of breast cancer (85). In summary, these studies identified ROR as an oncogene in human cancers and established it as a potential cancer biomarker.

### Antisense Non-Coding RNA in the INK4 Locus

ANRIL is a 3.8-kb-long transcript consisting of 19 exons (163). It is located on the human chromosome 9p21 locus, which contains three genes: CDKN2A (encoding p14^ARF^ and p16^INK4a^) and CDKN2B (encoding p15^INK4b^) (164). ANRIL was initially discovered in the hereditary cutaneous melanoma-neural system tumor syndrome family with a large germline deletion of the entire CDKN2A and CDKN2B gene cluster (163). ANRIL expression has been reported to be upregulated in many malignancies, such as colorectal (165), gastric (166), and brain cancers (167). Some studies have shown that the ectopic expression of ANRIL is associated with increased tumor size, advanced TNM stage, and poor clinical outcomes (168). Elevated ANRIL expression has been found in breast cancer, particularly in triple-negative breast cancer (86, 87). ANRIL was also included in a three-ncRNA signature, which was proposed to distinguish triple-negative breast cancer from other subtypes (87). ANRIL promotes tumorigenesis in triple-negative breast cancer by directly binding to miR-199a (88). In breast cancer, ANRIL was found to be predominantly located in the nucleus, and nuclear ANRIL positively correlated with periostin expression, suggesting that the subcellular localization of ANRIL impacts cancer progression (89). Moreover, ANRIL coordinates the expression of adjacent tumor-associated genes to promote carcinogenesis. ANRIL could bind to and recruit PRC2 to attenuate the expression of p15^INK4b^ (169). Furthermore, 9p21 polymorphisms have been implicated in cancer susceptibility. In breast cancer patients, the rs11515 CG genotype was more common and correlated with increased ANRIL expression and decreased p16^INK4a^ expression (90). Another study showed that ANRIL was linked to breast cancer susceptibility at the haplotype level and that haplotype analysis was more efficient than single nucleotide polymorphism (SNP) analyses (91). Hence, targeting ANRIL could provide novel insight into breast cancer treatment.

### Brain Cytoplasmic 200

BC200 is a 200-nucleotide-long transcript that is also known as brain cytoplasmic RNA 1 (BCYRN1) (170). BC200 is expressed exclusively to the nervous system, where it acts as a translational modulator (171). In 1997, abnormal expression of BC200 was found in diverse human cancers, such as breast, cervix, lung and ovary cancers (172). BC200 is overexpressed in proliferating cultured cells regardless of their origin. Knockdown of BC200 leads to decreased cell viability through regulation of growth arrest and induction of apoptosis (173). In non-small-cell lung cancer, BC200 increased the expression of matrix metalloproteases (MMPs), MMP-9 and MMP-13, resulting in enhanced invasion and migration (174). In addition, BC200 increased the expression of MMP-9 in colon cancer (175). In cervical cancer, BC200 competitively binds with miRNA-138, which leads to the enhancement of cell proliferation and metastasis (176). Moreover, elevated expression of BC200 has also been detected in luminal and triple-negative breast cancer cell lines. High BC200 levels could lead to increased cell viability, growth, migration, and invasion *in vitro* as well as to increased tumor size *in vivo* (177). Intriguingly, the expression of BC200 in ER-positive tumors was higher than that in ER-negative tumors. Mechanistically, BC200 binds to B-cell leukemia/lymphoma-x (Bcl-x) pre-mRNA to coordinate its alternative splicing, which results in suppressed expression of Bcl-xS and overexpression of Bcl-xL (92). Furthermore, BC200 RNA was reported to be significantly expressed in invasive breast cancer tissues but was not detectable in benign tumor tissues (93). Lacoangeli et al. also showed that plasma BC200 RNA levels were markedly elevated in invasive breast cancer patients compared to healthy subjects (94, 174), indicating that BC200 is a noninvasive molecular marker for invasive breast cancer detection.

### SPRY4 Intronic Transcript 1

SPRY4-IT1 is a 708-bp transcript located on chromosome 5 (95). It has specific secondary structures, which are possibly related to its functional properties (178). SPRY4-IT1 was initially identified as an oncogene in melanoma (178). To date, dysregulation of SPRY4-IT1 has been detected in multiple cancers, such as colorectal (179), non-small-cell lung (180), and breast cancers (96). Some studies have shown that upregulated SPRY4-IT1 expression decreased apoptosis and increased proliferation and migration (97). Expression profile analysis of breast cancer samples revealed that the expression of SPRY4-IT1 was upregulated, and SPRY4-IT1 had a good specificity value (96). Interestingly, the expression level of SPRY4-IT1 in ER‐negative tumors is higher than that in ER‐positive tumors, suggesting that estradiol expression may inversely correlate with SPRY4-IT1 expression (98). Functionally, deletion of SPRY4-IT1 induced G0/G1 cell cycle arrest and apoptosis of breast cancer cells by downregulating the expression of the oncogene zinc finger 703 (ZNF703) (98). Moreover, an N-terminal polypeptide derived from vMIP-II (NT21MP) downregulated SPRY4-IT1 expression, and the oncogenic role of SPRY4-IT1 was compromised by depletion of SKA2, suggesting that the antitumor activity of NT21MP was, at least partly, mediated through the SPRY4-IT1/SKA2 signaling pathway (99). Xiang et al. indicated that high SPRY4-IT1 levels correlated with increased tumor size, high TNM stage, lymph node metastasis and unfavorable clinical outcomes (181). The aforementioned findings indicate that SPRY4-IT1 may serve as a potential biomarker for the diagnosis and prognosis of breast cancer.

### Urothelial Carcinoma Associated 1

UCA1 is a 1,442-bp transcript located on chromosome 19p13.12 (182, 183). UCA1 was first identified in bladder cancer and is considered a novel oncogenic lncRNA (184). UCA1 is ubiquitously expressed in embryonic tissues but not in normal adult tissues except for the heart and spleen (185). UCA1 expression is significantly upregulated in many types of cancers, and high levels of UCA1 are associated with enhanced cell proliferation, invasion and metastasis (186). For instance, UCA1 was shown to promote both the proliferation and migration of lung cancer cells by targeting the miR-193a/HMGB1 axis (187). In addition, Luo et al. confirmed that UCA1 enhanced invasion and EMT by suppressing the expression of miR-143 in bladder cancer (188). Li et al. examined the strong association between UCA1 and protein tyrosine phosphatase 1B (PTP1B). Their results showed that the regulation of PTP1B by UCA1 was involved in the proliferation of breast cancer cells (100). Moreover, high expression of UCA1 activated the wnt/β‐catenin signaling pathway, enhanced the nuclear translocation of β‐catenin and promoted invasion in breast cancer. In addition, knockdown of UCA1 inhibited the EMT process by downregulating the expression of β‐catenin and its downstream targets MMP‐7 and cyclin D1 (101). It has also been shown that the lncRNA AC026904 and UCA1 cooperatively increase Slug expression at both the transcriptional and post-transcriptional levels, thereby inducing EMT and metastasis in breast cancer (102). Furthermore, it has been demonstrated that higher levels of UCA1 are associated with shorter OS and increased lymph node metastasis in multiple human cancers (103, 104).

### Activated by Transforming Growth Factor β

ATB is a 2,446-bp non-polyadenylated lncRNA located on human chromosome 14 (189). Numerous studies have evaluated the function of ATB in tumorigenesis. ATB was initially discovered as an oncogene in hepatocellular carcinoma, and high levels of ATB were associated with poor clinical outcomes (189). As a mediator of TGF‐β, ATB can regulate different transcription factors to induce the invasion-metastasis cascade. ATB was reported to be highly expressed in breast cancer tissues compared with non-cancerous tissues and the investigated cell lines, and this increase in ATB levels was associated with more nodal metastasis, advanced clinical stage and unfavorable prognosis (105, 106). In addition, the serum level of ATB was significantly elevated in breast cancer patients and could serve as a novel diagnostic biomarker for stage I-II breast cancer patients (107). Functionally, ATB increased the expression of Twist by sponging the miR‐200 family, consequently inducing EMT (105). Furthermore, downregulation of ATB expression could promote E-cadherin expression and suppress EMT by targeting miR-141-3p (106). Moreover, highly expressed ATB could act as a ceRNA for miR-200c and upregulate the expression of the miR-200c target genes ZEB1 and ZNF-217 to promote invasiveness and trastuzumab resistance in HER2-positive breast cancer (108). Intriguingly, the oncogenic role of ATB has been disputed by conflicting studies. For instance, ATB acts as a tumor suppressor in pancreatic cancer (190). Similarly, Nikpayam et al. showed that ATB expression was significantly downregulated in most breast cancer tissues compared with adjacent non-cancerous tissues (109). Both upregulation and downregulation of ATB expression have been indicated to contribute to tumorigenesis, suggesting that ATB might play distinct roles in different cancers or even different cancer subtypes. Further mechanistic studies should be focused on elucidating the role of ATB in cancer pathology.

### Plasmacytoma Variant Translocation 1

PVT1, an intergenic lncRNA, is located on chromosome 8q24.21 adjacent to c-Myc (191). PVT1 is highly expressed in cancer tissues compared with non-cancerous tissues and in cancer cell lines (192, 193). Co-amplification of adjacent PVT1 and Myc has been found in many human cancers. PVT1 increases Myc protein levels in 8q24-gain cancers, while either Myc or PVT1 fails to measurably promote cancer (194). Moreover, depletion of PVT1 resulted in decreased c-Myc expression and increased apoptosis of cancer cells (195). PVT1 could also enhance the stability of Kruppel-like factor 5 (KLF5) and increase the expression of β-catenin, an important downstream effector of KLF5, to promote tumorigenesis in triple-negative breast cancer (110). Several studies have shown the connection between PVT1 and different miRNAs in breast cancer. PVT1 functions as a sponge to regulate miR-543 (111), which is a known tumor suppressor miRNA in breast cancer (112). The tumorigenic potency of PVT1 could be abrogated by miR-543 overexpression, and loss of PVT1 is associated with inhibition of growth, increased apoptosis, and decreased tumor size (111). A cluster of oncogenes (miR-1204, 1205, 1206, 1207-3p, 1207-5p, 1208) at the 8q24.21 locus is regulated by PVT1 (193). For instance, PVT1 upregulates the expression of miR-1207-5p to repress the expression of signal transducer and activator of transcription 6 (STAT6) and cyclin inhibitors, thus enhancing cell proliferation and colony formation in breast cancer (113). Additionally, miR-1204 overexpression contributes to the proliferation, invasion and EMT of breast cancer cells both *in vitro* and *in vivo* (114). Furthermore, a meta-analysis carried out by Lu et al. indicated that high PVT1 expression correlated with more lymph node metastasis, increased distant metastasis, advanced TNM stage, poor differentiation grade and unfavorable prognosis but not with tumor volume (115). Thus, PVT1 could act as a useful molecular marker for breast cancer.

### Colon Cancer Associated Transcript 1

CCAT1, initially identified in colon cancer, is mapped to the 8q.24.2 locus and is ~2,628 nucleotides long (196). The 8q.24.2 locus contains only a few protein-coding genes and is often referred to as a ‘gene desert’ (197). CCAT1 expression is consistently upregulated in multiple types of cancers and correlates with poor prognosis (198). Han et al. found that CCAT1 was overexpressed in triple-negative breast cancer tissues compared to adjacent normal tissues and in a panel of triple-negative breast cancer cell lines in comparison to normal breast epithelial cell lines (116). CCAT1 has been shown to act as a decoy to inhibit the expression of several miRNAs. Loss of CCAT1 resulted in the upregulation of miR-218 expression and the simultaneous inhibition of a miR-218 target gene, zinc finger protein ZFX, resulting in inhibited cell proliferation, migration, and invasion. Moreover, silencing of miR-218, in turn, can block the tumor suppressive effect of CCAT1 knockdown, suggesting that CCAT1 may promote breast carcinogenesis through regulation of the miR-218/ZFX axis (116). Another study showed that the expression of CCAT1 was higher in radioresistant breast cancer tissues than in radiosensitive breast cancer tissues. Depletion of CCAT1 dramatically decreased the colony formation rate and promoted apoptosis by directly interacting with miR-148b. The authors concluded that loss of CCAT1 might enhance the radiosensitivity of breast cancer cells by downregulating miR-148b expression (117). CCAT1 could function as a regulator of wnt/β-catenin signaling pathway in cervical cancer (199) and non-small-cell lung cancer (200). Consistent with these studies, Tang et al. indicated that CCAT1 coordinated miR-204/211, miR-148a/152 and annexin A2 to hyperactivate the wnt/β-catenin signaling pathway, consequently promoting the proliferation and metastasis of breast cancer stem cells (118). Overexpression of CCAT1 in breast cancer has been related to histological grade, TNM staging and lymph metastasis, and it is also an independent predictor of OS and progression-free survival (PFS) (119). The ubiquitous nature of CCAT1 upregulation in cancers shows promise for future discovery of diagnostic biomarkers and pharmaceutical targets for cancer control.

### Colon Cancer Associated Transcript 2

CCAT2 is 1,752 bp in length and is located within the chromosome 8q24.21 gene desert adjacent to Myc (201). Amplification of the oncogenes in the 8q24.21 region has been found in numerous human cancers. High CCAT2 levels positively correlated with Myc levels in colon and colorectal cancer (202, 203). Accordingly, CCAT2 could upregulate the expression of Myc in breast cancer, suggesting that the amplification of CCAT2 and Myc might occur simultaneously (120). Huang et al. explored the expression of CCAT2 in 33 cancer types and 13,285 tumor patients. The study revealed that CCAT2 was substantially overexpressed in cancer tissues compared to paired normal tissues, and this increase in CCAT2 levels correlated with a greater tumor volume, higher TNM grades, advanced clinical stage and a poor OS in patients. In addition, CCAT2 expression was mainly upregulated in stage II tumor pathology, followed by stage III, indicating that CCAT2 could be used for the early detection of cancers (121). Moreover, CCAT2 expression levels in metastatic breast cancer were higher than those in non-metastatic breast cancer. Downregulation of CCAT2 expression significantly inhibited the expression of TGF-β, Smad2 and α-SMA, thereby inducing apoptosis and G0/G1 cell cycle arrest (122). Deng et al. indicated that CCAT2 knockdown suppressed the expression of cell cycle-related proteins and G0/G1 phase arrest in breast cancer cells (204). They also found that CCAT2 interacted with EZH2, a marker of aggressive breast cancer (123) and abrogated the expression of P15 (204). It has been shown that Notch signaling could be activated and upregulated in breast cancer (205). Xu et al. demonstrated the strong association between CCAT2 and Notch 2 in triple-negative breast cancer (124). Functionally, CCAT2 promoted the growth, invasion and migration of breast cancer stem cells by sponging miR-205, which targets Notch 2 (124). Overall, accumulating evidence suggests that CCAT2 is an oncogene and could serve as a useful biomarker and therapeutic target for breast cancer treatment.

### Tissue Differentiation-Induced Non-Coding RNA

TINCR is highly expressed in keratinocytes and is essential for normal epidermal differentiation (206). It is a 3,733-nucleotide long transcript located on chromosome 19p13 (207). Aberrant TINCR expression has been implicated in multiple human cancers. TINCR expression is upregulated in gastric, gladder and breast cancer but downregulated in glioma and prostate cancer (208). In recent years, several studies have been performed on the contribution of TINCR to breast cancer. Liu et al. indicated that TINCR was activated by transcription factor specificity protein 1 (SP1) in breast cancer (125). Consistent with this observation, Xu et al. showed that SP1 could bind to the putative GC-rich motifs of TINCR to upregulate the expression of TINCR in gastric cancer (209). In addition, TINCR overexpression competed with miR-7 and facilitated KLF4 expression, which in turn regulated cell proliferation, migration, and invasion in breast cancer (125). Insulin-like growth factor receptor 1 (IGFR-1), a tyrosine kinase cell surface receptor, is involved in the development and progression of breast cancer (210). Guo et al. showed that TINCR played an oncogenic role in breast cancer through regulation of the miR-589-3p/IGF1R axis (126). Moreover, the expression of TINCR was higher in trastuzumab-resistant tissues than in sensitive tissues owing to enhanced histone acetylation of the TINCR promoter. Functionally, TINCR promoted the expression of HER-2 by sponging miR-125b, consequently conferring trastuzumab resistance (127). Moreover, TINCR promoted EMT *via* downregulation of Snail-1 expression, while enhanced Snail-1 expression reversed EMT suppression induced by TINCR silencing in trastuzumab-resistant cell lines (127). Furthermore, Kaplan-Meier survival curves showed that high levels of tissue TINCR correlated with unfavorable prognosis in breast cancer (126). Wang et al. found that circulating TINCR was dramatically elevated in breast cancer, particularly in the aggressive triple-negative subtype. The authors further noted that serum TINCR levels were associated with unfavorable prognosis, suggesting that TINCR could serve as a novel biomarker for breast cancer therapy (128).

## Tumor Suppressor LNCRNAS

### Maternally Expressed Gene 3

MEG3 is an imprinted gene from the maternal allele mapped to the human chromosome 14q32.3 region (211). The transcript contains 10 exons and approximately 12 alternative splicing isoforms, some of which are expressed in a tissue- or cell-type-specific manner (211). MEG3 was the first lncRNA to be identified as a tumor suppressor in the inhibition of cancer cell growth (212). A loss of MEG3 expression has been found across human cancer cell lines, and decreased MEG3 levels significantly correlate with TNM stage, lymph node metastasis and differentiation grade (129, 130). Loss of MEG3 expression also predicts shorter OS, PFS, distant metastasis-free survival (DMFS), and disease-specific survival (DSS) (130–132). Zhang et al. showed that ectopic MEG3 overexpression promoted breast cancer progression by upregulating the expression of the endoplasmic reticulum stress-related proteins NF−κB and p53 (133). Mechanistically, MEG3 can bind directly to the p53 promoter and increase the transcriptional activity of p53, thus regulating the expression of p53 target genes (134). In addition, MEG3 deactivated the AKT/mTOR signaling pathway by sponging miR-21, while miR-21 overexpression partially abolished the tumor suppressive function of MEG3 in breast cancer cells (135). Moreover, elevated expression of MEG3 can inhibit cell invasion, proliferation, and apoptosis induction (213, 214), indicating that MEG3 might be a novel therapeutic target for cancers. SNPs mainly refer to a set of DNA sequence polymorphisms based on single nucleotide variations at the genomic level (215). It has been reported that SNPs are linked to genetic susceptibility to cancer (216). Ali et al. indicated that MEG3 rs7158663 G > A with the mutant A allele correlated with decreased serum MEG3 expression and unfavorable clinical outcomes in an Egyptian population (136). Additionally, the GG genotype of rs3087918 could influence the secondary structure of MEG3 and decrease the susceptibility to breast cancer risk in Chinese women (137). Hence, MEG3 could be a suitable biomarker candidate for clinical cancer management.

### X-Inactive Specific Transcript

XIST, 17 kb in length, is located at the X-inactivation center (217). During primary embryogenesis, XIST recruits multiple factors to orchestrate X chromosome inactivation (218). Recent studies have identified associations between aberrant XIST expression and breast cancer. Zheng et al. indicated that XIST expression was drastically downregulated in breast cancer tissues and cell lines. The authors also found that XIST sponged miR-155, which in turn upregulated the expression of caudal-type homeobox 1 (CDX1) and inhibited the progression of breast cancer (138). In addition, Liu et al. also found that XIST functioned as a ceRNA for miR-362-5p and thus inhibited its repressive effect on ubiquitin-associated protein 1 (UBAP1), consequently inhibiting breast cancer progression (139). BRCA1 is a high-penetrance gene in which loss-of-function mutations predispose patients to breast and ovarian cancers (140, 141). Sirchia et al. indicated that BRCA1 participates in XIST regulation on the active X chromosome as well as XIST dysregulation and drives tumorigenesis in breast cancer. Mechanistically, BRCA1 knockdown resulted in enhanced XIST expression, promoter demethylation and X chromosome inactivation (142). However, another study suggested the potential oncogenic role of XIST in breast cancer. Zong et al. showed that XIST knockdown dramatically reduced characteristics associated with breast cancer, such as cell proliferation, anti-apoptosis, invasion, and migration activities. Functionally, XIST induced sponging of miR-125b-5p and removed the inhibitory effect of this miRNA on NLRC5, a breast cancer promotor, thus promoting the malignancy of breast cancer cells (143). In addition, a meta-analysis carried out by Zhu et al. demonstrated that XIST was overexpressed in multiple cancers and that elevated XIST levels correlated with larger tumor volume, increased lymphatic metastasis, advanced tumor stage and unfavorable clinical outcomes (144). XIST could serve as an oncogene or tumor suppressor, and further studies are still needed to elucidate the roles of XIST in cancer biology.

### Growth Arrest−Specific Transcript 5

GAS5, a well-known tumor suppressor, is located on chromosome 1q25 (219). Abnormal expression levels of GAS5 have been reported in different cancer types (220–222). For example, GAS5 has been shown to promote proliferation by regulating miR-22 and its downstream target transcripts in gastric cancer (223). GAS5 can also promote cell invasion and migration by targeting miR-196a and the PI3K/Akt/mTOR signaling pathway in oesophageal squamous cell carcinoma (224, 225). In ovarian cancer, loss of GAS5 is related to increased tumor volume and advanced tumor stage (226, 227). In addition, the expression of GAS5 is significantly downregulated in breast cancer tissues compared with adjacent non-cancerous tissues (228). Larger tumor volume, advanced lymph node metastasis, and estrogen receptor negativity in breast cancer cells are the outcomes of GAS5 downregulation (229). In HER2-positive breast cancer, silencing of GAS5 contributes to trastuzumab resistance. Mechanistically, GAS5 serves as a molecular sponge of miR-21 to increase the expression of phosphatase and tensin homologs (PTEN) and alleviate trastuzumab resistance (230). Zhang et al. demonstrated the reciprocal inhibition between miR-21 and GAS5 in breast cancer. MiR-21 downregulated GAS5 expression, while silencing of GAS5 increased miR-21 expression (231). Jing et al. found that GAS5 expression was significantly downregulated by Notch‐1 and that decreased GAS5 levels were involved in the proliferation of breast cancer (232). Thus, these studies demonstrate that GAS5 could be an attractive biomarker candidate in cancer therapy.

### Phosphatase and Tensin Homolog Pseudogene 1

As a pseudogene of PTEN, PTENP1 has a highly homologous region upstream of the 3′UTR of PTEN (233). To date, aberrant expression of PTENP1 has been found in various malignancies, including breast cancer (73). Low levels of PTENP1 have been shown to be associated with increased proliferation, migration, invasion and colony formation, as well as decreased apoptosis, in breast cancer (73, 145, 146). PTENP1 has been implicated in the regulation of the PI3K/Akt signaling pathway, which plays a pivotal role in tumorigenesis and tumor development, particularly in breast cancer (147). Chen et al. indicated that PTENP1 suppressed breast cancer cell proliferation and migration *via* regulation of Akt and cell cycle-related proteins (145). In addition, PTENP1-induced sponging of miR-19b resulted in increased expression of PTEN (73, 146) and decreased expression of p-PI3K, PI3K and p-Akt, thereby inhibiting cell proliferation and migration (73). Moreover, the regulatory effect of PTENP1 on the PI3K/Akt signaling pathway can be reversed by the overexpression of miR-19b (73). Furthermore, Gao et al. showed that PTENP1 inversely correlated with miR-20, a known oncogenic mRNA. PTENP1 acts as a decoy for miR-20 to derepress its inhibitory effect on PTEN, ultimately attenuating the activation of the PI3K/Akt pathway (148). They also found that low expression of PTENP1 and PTEN was associated with advanced TNM stage and worse OS (148). Interestingly, the involvement of PTENP1 in breast cancer biology may depend on the hormone receptor status. PTENP1 overexpression was linked to decreased PTEN expression and increased proliferation in ER-positive cells, while increased PTEN expression and inhibited tumorigenesis were observed in ER-negative cells (149). Hence, PTEN may represent a promising biomarker for breast cancer.

## Circulating LNCRNAS as Biomarkers in Cancer

Biomarker is defined as “a characteristic that is objectively measured and evaluated as an indicator of normal biological processes, pathogenic processes or pharmacologic responses to a therapeutic intervention” by the US NIH’s Biomarkers Definition Working Group and the Biomarkers Consortium (1). A tumor marker is any specific molecule indicating the presence or progression of human cancers. Tumor biomarkers can be either found in body fluids or tumor tissues. Biomarkers in body fluids (especially those in blood serum) are readily measured, and their diagnostic performances have been confirmed in multiple cancers. Carcinoembryonic antigen (CEA) and cancer antigen 15-3 (CA15-3) in serum have been approved by the US Food and Drug Administration (FDA) as biomarkers for breast cancer. Notwithstanding CEA and CA15-3 are widely used in diagnosis of breast cancer, they bear some limitations, mainly regarding to low sensitivity and specificity. Therefore, it is critical to discover novel molecular markers with improved diagnostic value.

Dysregulated lncRNAs in primary tumor tissues could be mirrored in different body fluids, such as blood plasma, urine and saliva (234–236). Many studies have revealed that lncRNAs remain stable while circulating in body fluids even under extreme conditions, further enhancing their competitive advantage of being good diagnostic tools. In recent years, several circulating lncRNAs have been proved as suitable diagnostic and prognostic markers in various cancer types, such as prostate cancer antigen 3 (PCA3) and MALAT1. lncRNA PCA3 in urine samples has received the approval of the FDA as a diagnostic molecule for prostate cancer. Intriguingly, lncRNA PCA3 is much more specific and sensitive than prostate-specific antigen, the conventional gold standard for prostate cancer. A meta-analysis carried out by Xue et al. has determined the diagnostic value of PCA3 for the detection of prostate cancer, with sensitivity and specificity of 62% and 75%, respectively (235). Also, plasma H19 holds great potential as an independent biomarker for gastric cancer due to its high diagnostic performance (sensitivity 82.9%; specificity 72.9%) (234). In addition, serum MALAT1 has proven its diagnostic value for breast cancer (sensitivity 83.7%; specificity 81.2%) (237). More importantly, lncRNA-based detection method is noninvasive, convenient and inexpensive when compared to the traditional biopsies.

## Prospects and Challenges

Since lncRNAs are dysregulated in cancers, the functional lncRNAs may be targeted to halt the process of carcinogenesis. LncRNAs targeting strategies can be achieved by antisense oligonucleotides (ASOs), RNA interference (RNAi) and clustered regularly-interspaced, CRISPR-Cas9, etc. It was suggested that ASOs mainly targeted the lncRNAs retaining in the nucleus, whereas RNAi predominantly targeted the lncRNAs in the cytoplasm (238). CRISPR-Cas9, a precise versatile toolkit, could target lncRNAs at high throughput, representing a major technology breakthrough in gene editing (239).

Although lncRNAs hold potential to serve as ideal diagnostic biomarkers and therapeutic targets, some challenges need to be addressed and resolved in the future. First, the poor consequence conversation of lncRNAs has complicated the pre-clinical studies across different species. In addition, lncRNAs have secondary and tertiary structures, which could lead to ineffectiveness of the lncRNAs targeting therapeutics. Secondly, the lncRNA-based treatment may perturb other genes. Thus, there is a risk of off-target effects and new strategies should be developed to maximize the on-target efficacy. Thirdly, the concentration of circulating lncRNAs may be below the detection limit of the existing equipment, such as NanoDrop spectrophotometer. It is expected that in the near future, more sensitive detection instruments will open a new window for lncRNA quantification. Fourthly, a single lncRNA may not be feasible for cancer diagnosis. Xie et al. suggested that a diagnostic panel for NSCLC possessed higher specificity (79.2%) and sensitivity (77.1%) when compared to any single molecular marker, such as CEA and lncRNA ANRIL (240). Indeed, biomarkers in a panel can complement each other, contributing to enhanced diagnostic performance.

## Concluding Remarks

As indicated in this review, lncRNAs have gained considerable attention as pivotal regulators in various physiological and pathophysiological events. Altered expression levels of lncRNAs have been reported in multiple human cancers, including breast cancer. It has become clear that lncRNAs with dysregulated expression drive the initiation and progression of cancers *via* interactions with other types of RNA molecules, DNA and proteins. Intriguingly, lncRNAs are differentially regulated in diverse cancers or even cancer subtypes and show a significant association with pathological features and clinical prognosis. Regarding the aberrant expression of lncRNAs and the underlying mechanisms, lncRNAs may act as suitable diagnostic and prognostic biomarkers in breast cancer. Furthermore, lncRNAs could be targeted to reverse the process of carcinogenesis and represent valuable therapeutic targets for cancer treatment. LncRNA-based tests and therapy are promising strategies that deserve extensive research and thorough exploration in the future.

## Author Contributions

WZ and CL were responsible for the study conception and design. CL drafted the manuscript. DW and PW contributed to the significant portions of the manuscript. WZ and YZ revised and edited the manuscript. All authors contributed to the article and approved the submitted version.

## Funding

This study was supported by the National Natural Science Foundation of China (Grant No. 81800643).

## Conflict of Interest

The authors declare that the research was conducted in the absence of any commercial or financial relationships that could be construed as a potential conflict of interest.

## Publisher’s Note

All claims expressed in this article are solely those of the authors and do not necessarily represent those of their affiliated organizations, or those of the publisher, the editors and the reviewers. Any product that may be evaluated in this article, or claim that may be made by its manufacturer, is not guaranteed or endorsed by the publisher.
